# Prevalence and factors associated with depression among health care workers in the region of Sousse in Tunisia

**DOI:** 10.1192/j.eurpsy.2023.1271

**Published:** 2023-07-19

**Authors:** Z. Athimini, A. Gaddour, A. Brahem, R. El Ghezhal, A. Chouchene, M. Bouhoula, O. Elmaalel, S. Chatti, N. Mrizak

**Affiliations:** 1 occupational medicine departement, Farhat Hached University Hospital; 2 occupational medicine departement, university hospital of kairouan in Tunisia; 3 psychiatry department, Farhat Hached University Hospital, sousse, Tunisia

## Abstract

**Introduction:**

Mental health disorder is common among working population worldwide and among health care-workers (HCWs) in particular. Depression is a major public health problem, with an economic impact because of lost days of work. Its prevention is essential and requires the identification of risk factors.

**Objectives:**

The objectives of this work were to determine the epidemiological characteristics of depressive disorders in health care workers and identify their main risk factors.

**Methods:**

A descriptive retrospective study was conducted on health care workers of Sousse in Tunisia who have had a long-term sick leave for depressive disorders from January 2010 to December 2021. data was collected from the medical records of the patients and completed with a telephone questionnaire

**Results:**

The total number of cases was 650 with a prevalence of 12.8% and an incidence of 2 cases per 100 HCW. The median age was 50 years and the female workers represented 81% of cases. The majority of the sample were married (81%). Most of cases were nurses (43%) and health technician (19%). The median seniority of HCW in their jobs was 23 years with the first quartile at 12 and the third quartile at 31.

Around 48% of cases had severe depression. The severity of depression was significantly associated with working in surgical and emergency services, number of night shifts of 2 or more per week, the history of a psychiatric disorder other than depression, habits such as smoking and drinking, anxiety specificity and melancholy specificity of depression.

**Conclusions:**

This study showed the importance of social and occupational factors of depression among HCW. Action policies focusing on workplace interventions appear to be relevant.

**Disclosure of Interest:**

None Declared

